# Approach to Sudden Hearing Loss Among Primary Care Physicians in Riyadh, Saudi Arabia

**DOI:** 10.7759/cureus.55849

**Published:** 2024-03-09

**Authors:** Nader F Aldajani, Abdulrahman M Aloufi, Nujud A Binhudayb, Buthaina J Yahya, Abdullah F Alkarni

**Affiliations:** 1 Otolaryngology-Head and Neck Surgery, King Fahad Medical City, Riyadh, SAU; 2 Clinical Sciences, College of Medicine-Almaarefa University, Riyadh, SAU; 3 Otolaryngology-Head and Neck Surgery, King Abdulaziz Medical City Riyadh, Riyadh, SAU

**Keywords:** tuning fork test, rinne test, weber test, conductive hearing loss, primary care physicians, sudden sensorineural hearing loss

## Abstract

Introduction: A medical emergency known as sudden sensorineural hearing loss (SSNHL) affects the ears suddenly, has a considerable probability of negative cognitive and functional outcomes, and can influence the patient's quality of life. Primary care physicians play a crucial role in diagnosing SSNHL and initiating prompt and efficient management since they are the ones who would likely encounter it initially. This study aims to evaluate the present knowledge, diagnostic, and management perspective of SSNHL among primary care physicians in Riyadh, Saudi Arabia.

Methods: A self-generated questionnaire with 17 questions was developed, and a link to the online survey was delivered to primary care physicians (PHPs) in Riyadh, Saudi Arabia, concerning the management of SSNHL.

Results: The knowledge level regarding SSNHL was evaluated, in which 21 (25%) of the participants had a low knowledge level, 34 (40.5%) had moderate knowledge, and 29 (34.5%) had a high knowledge level. Among 84 participants, 20 (23.8%) were confident in their ability to administer and understand the findings of tuning fork tests (TFT) to differentiate between sensorineural hearing loss and conductive hearing loss, whereas 64 (76.2%) were unsure about it. In addition, to distinguish between sensorineural hearing loss and conductive hearing loss, 62 (73.8%) participants were confident, and 22 (26.2%) participants were skeptical about their ability to interpret a formal audiogram.

Conclusion: Considering SSNHL as a medical emergency, in our survey, many family doctors would make proper referral and treatment decisions. However, TFTs were underutilized for guiding management decisions compared to other ways to distinguish between conductive and sensorineural hearing loss.

## Introduction

A condition known as sudden sensorineural hearing loss (SSNHL) results in hearing loss within 72 hours after commencement [1]. SSNHL requires immediate attention because delayed treatment could result in long-term deafness and detrimental effects [2]. This can occur at any age but is most frequent in those aged 65 years and older [3]. The global incidence rate of SSNHL is quoted as 5-20 cases per 100,000 people per year [4]. In Saudi Arabia, 1-4 cases were found in 1000 people [5].

Most SSNHL cases are idiopathic, but they could be attributed to vascular traumas, infections, autoimmune illnesses, cerebellopontine cell tumors, or inner ear malformations [6]. When an inactive neurotropic virus in the inner ear invades or is reactivated, it damages the cochlea or cochlear nerve, resulting in SSNHL. On the other hand, a systemic viral infection can cause this disorder by stimulating an immune-mediated response or stress-signaling pathway in the cochlea [7]. Furthermore, acute vascular hemorrhage, insufficient blood supply to the cochlea, embolic blockage, vascular disease, vasospasm, or hyperviscosity can also result in SSNHL [8-14]. Besides several autoimmune diseases, including Cogan’s syndrome [15-16], cerebral palsy, gout, lupus, and Behçet syndrome can also result in this ailment [15-23].

Reports indicate that severity and type of hearing loss at the time of onset have predictive significance. Patients who suffer from a more serious hearing impairment when they first arrive are more likely to have a difficult recovery [24-26]. Flat or downward-sloping audiogram shapes are associated with a poor prognosis, whereas upward-sloping audiograms can indicate a better prognosis. It is suggested that low-frequency or mid-frequency hearing loss tends to have greater recovery rates because of better impairment tolerance [27-28].

Primary care physicians (PCPs) are responsible for identifying prospective cases and initiating proper management as promptly as possible because they are typically the initial contact for people acquiring SSNHL in the healthcare setting. There is a dearth of studies examining how Saudi Arabian PCPs manage patients who experience unexpected hearing loss. SSNHL is still a complex and challenging process regarding its etiology, therapy, and prognostic aspects. Considering the variation and management's ambiguity in SSNHL [29]. This study aims to assess current knowledge, diagnostic tools, and management of SSNHL among PCPS within Riyadh, Saudi Arabia. This would also provide insight into the gaps in PCPs' education and practice that would be addressed to improve patient care and outcomes.

## Materials and methods

Study setting and sample size

This cross-sectional study was carried out in primary healthcare facilities in Riyadh, Saudi Arabia, to assess the management of SSNHL. The data were gathered through an online questionnaire from family physicians in primary health care centers. The study and data collection duration started in May 2022 and ended in July 2022. The inclusion criteria were as follows: primary health care family physicians, residents, fellows, and consultants in all family medicine subspecialties. We excluded medical interns, general practitioners, and other specialties in PHC centers. A sample of 84 PCPs was selected. The sample size calculation was based on the following considerations: the standard normal distribution value at a 95% confidence level of 1.96 and the margin of error (d) of 5%.

Study instruments

A self-administered questionnaire consisted of two sections: demographics of physicians and assessment questions regarding SSNHL. In the first section, the demographic characteristics of participants were asked, such as the number of years of physician practice and the practice setting. However, no information was collected that could be used to identify participants. In the second section, to evaluate current understanding and practice in SSNHL diagnosis and treatment, 12 multiple-choice questions were developed. All questions are scored from 0 to 17, with only one correct answer. The electronic questionnaire was circulated among PCPs through social networking sites, e.g., WhatsApp and Facebook groups, with PCPs as the only members. An introduction to the study and an information letter were distributed at the beginning of the survey to ensure that responses were only gathered from the intended demographic. Consent from participants was obtained before the commencement of the study. The questionnaire is available in the appendices. Permission was not required to use this questionnaire. It was based on the questionnaire developed by Ng et al. [2]. 

Statistical analysis

All the data obtained were reviewed for completeness and consistency before being input into a statistical package for the social sciences. IBM Corp. Released 2015. IBM SPSS Statistics for Windows, Version 23.0. Armonk, NY: IBM Corp. was used for data analysis. For categorical values, frequency and percentage were employed as displays. Numerical variables were presented as minimum, maximum, and mean values.

Ethical considerations

Before initiating data collection, the present study obtained ethical approval from the Committee of Research Ethics at Almaarefa University, IRB Approval No. IRB-23-073. Written consent was provided by all participants, which elucidated the study's purpose, significance, and privacy concerns. Confidentiality and anonymity of participants' data were safeguarded, and they retained the liberty to refuse or withdraw from the study at any juncture. No inducements or rewards were offered to the participants.

## Results

Participant demographics

**Figure 1:**
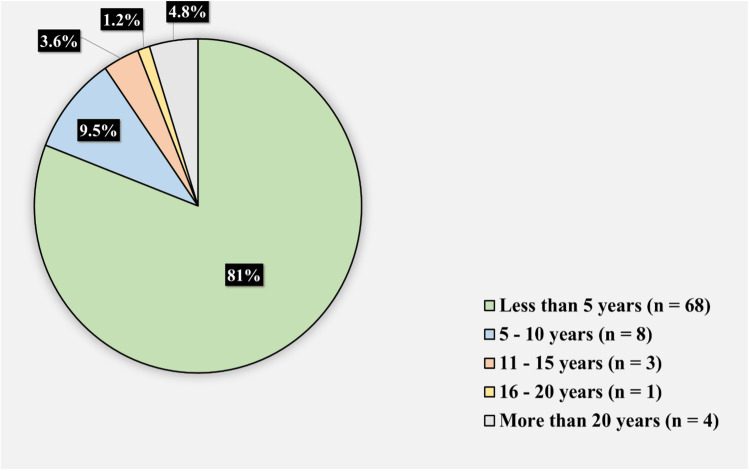
Participants' years in practice

The study included 84 individuals in total. Figure 1 shows the participants’ years of practice. Sixty-eight (81%) participants had been in practice for less than five years, eight (9.5%) had been in practice for 5-10 years, 3 (3.6%) had been in practice for 11-15 years, one (1.2%) had been in practice for 16-20 years, and four (4.8%) had been in practice for more than 20 years. About 47 (56%) participants reported to be working in walk-in clinics, 38 (45.2%) reported working in an academic group or team, 31 (36.9%) reported having solo practice, 20 (2.3%) reported being in an urgent care or emergency department, and eight (9.5%) reported being in a non-academic group or team. 

**Figure 2:**
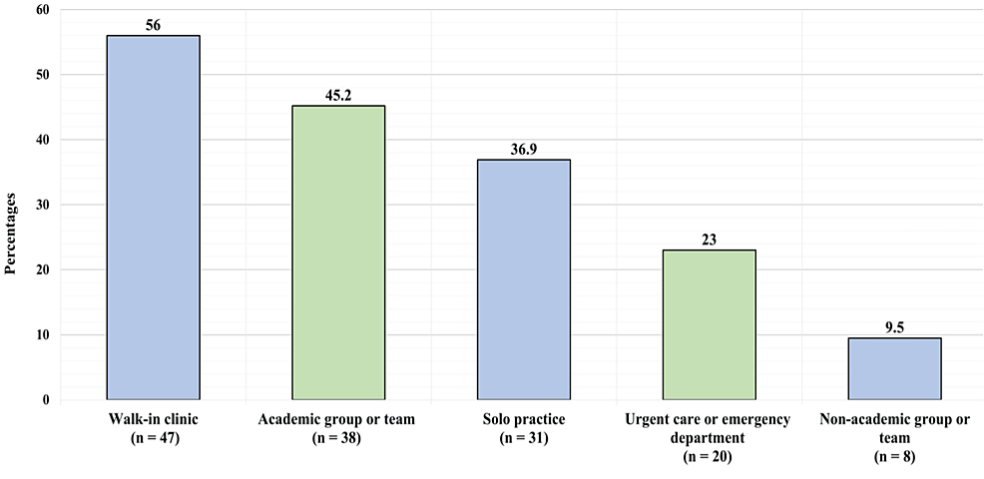
The setting in which your practice would be considered (more than one can be chosen)

Figure 2 presents the hospital in which participants’ primary practice takes place. Of 84 respondents, 41 (48.8%) reported working in King Fahad Medical City, and nine (10.7%) reported working in King Saud Medical City. Others reported working at different institutions in Riyadh, either private or governmental.

Knowledge and practice among PHPs

The participant's practice and experience profile with hearing loss are shown in Table 1.

**Table 1:** Participants practice and experience profile toward hearing loss (n = 84)

In the past 6 months, the number of patients presenting to you with complaints of unilateral, acute, or sudden- onset hearing loss, not as a result of cerumen impaction:		
Fewer than 5	73	86.90
5 - 10	6	7.10
11 - 15	4	4.80
More than 20	1	1.20
In the past 12 months, the typical wait-time for your patients to be seen by an otolaryngologist referred for unilateral, sudden-onset hearing loss:		
1 week or less	11	13.10
1 - 4 weeks	19	22.60
1 - 3 months	15	17.90
3 - 6 months	12	14.30
Greater than 6 months	3	3.60
I don't know	24	28.60
Do you feel confident in administering and interpreting the results of tuning fork tests to differentiate between conductive hearing loss and sensorineural hearing loss?		
Yes	64	76.2
No	20	23.8
Do you feel comfortable interpreting a formal audiogram to differentiate between conductive hearing loss and sensorineural hearing loss?		
Yes	62	73.8
No	22	26.2

When asked about the number of patients presenting with complaints of unilateral, acute, or sudden-onset hearing loss not as a result of cerumen impaction in the past six months, 73 (86.9%) reported it was fewer than five patients, six (7.1%) reported it was 5-10 patients, four (4.8%) reported it was 11-15 patients, and one (1.2%) reported it was more than 20 patients. As for the typical wait time for patients to be seen by an otolaryngologist referred for unilateral, sudden-onset hearing loss in the past 12 months, 11 (13.1%) reported it was one week or less, 19 (22.6%) reported it was 1-4 weeks, 15 (17.9%) reported it was 1-3 months, 12 (14.3%) reported it was 3-6 months, three (3.6%) reported it was greater than six months, and 24 (28.6%) reported they did not know. About 63 (75%) participants claimed to employ the Tuning Fork Test (TFT) to differentiate between sensorineural and conductive hearing loss. Twenty (23.8%) participants reported they were not confident, while 64 (76.2%) participants reported they were confident in their ability to administer and interpret the findings of tuning fork tests to differentiate between sensorineural hearing loss and conductive hearing loss. In addition, 62 (73.8%) participants stated they were confident in their ability to distinguish between sensorineural hearing loss and conductive hearing loss using a formal audiogram, whereas 22 (26.2%) participants indicated they were not confident.

Table 2 illustrates the assessment of participants’ knowledge toward sudden sensorineural hearing loss. The minimum knowledge score was three, the maximum was 17, and the mean was 10.89 + 3.24. Among the 84 participants, only 16 (19%) physicians claimed to prescribe corticosteroids as a treatment for suspected unilateral SSNHL. 

**Table 2:** Assessment of participants knowledge toward sudden sensorineural hearing loss (n = 84)

Question		n	%
According to your definition, sudden sensorineural hearing loss (SSNHL) is defined as hearing loss that can develop over a period of			
Less than 24 hours		33	39.3
48 hours		14	16.7
72 hours (correct answer)		22	26.2
More than 7 days		15	17.9
Which of the following referrals do you make upon presentation of suspected unilateral SSNHL*? "Check all that apply".			
Audiological evaluation (correct answer)		73	86.9
Otolaryngology consultation (correct answer)		56	66.7
Magnetic Resonance Imaging (MRI)		34	40.5
Computed Tomography (CT)		23	27.4
Emergency Department		23	27.4
Lab work		19	22.6
Neurology consultation		16	19.0
In your practice, does unilateral, sudden-onset hearing loss warrant urgent referral for audiological testing?			
Yes (correct answer)		72	85.7
No		12	14.3
In your practice, does unilateral SSNHL* warrant urgent referral to otolaryngology?			
Yes (correct answer)		71	84.50
No		13	15.50
When presented with unilateral, acute or sudden-onset hearing loss, do you attempt to differentiate between conductive and sensorineural hearing loss?			
Yes (correct answer)		71	84.5
No		13	15.5
Do you use tuning fork tests to differentiate between conductive and sensorineural hearing loss?			
Yes (correct answer)		63	75
No		21	25
When presented with unilateral, sudden-onset hearing loss, which of the following do you use to inform management decisions? "Check all that apply".			
Case history (correct answer)		75	89.3
Audiological evaluation (correct answer)		73	86.9
Tuning fork test(s) (correct answer)		65	77.4
Otoscope (correct answer)		63	75.0
Lab work		21	25.0
As a family physician, which of the following pharmacologic agents do you prescribe as treatment when presented with suspected unilateral SSNHL*, prior to confirmation with audiological testing? "Check all that apply".			
Corticosteroids (correct answer)		16	19.0
Antivirals		6	7.1
Vasodilators		6	7.1
Other (e.g., antibiotics)		4	4.8
Thrombolytics		0	0.0
None of the above		9	10.7
I do not prescribe any pharmacologic agents when presented with suspected SSNHL		59	70.2
As a family physician, which of the following pharmacologic agents do you prescribe as treatment when presented with confirmed, unilateral SSNHL*? "Check all that apply".			
Corticosteroids (correct answer)		22	26.2
Antivirals		5	6.0
Other (e.g., antibiotics)		4	4.8
Thrombolytics		3	3.6
Vasodilators		3	3.6
I do not prescribe any pharmacologic agents when presented with suspected SSNHL		44	52.4
None of the above		16	19.0
Which of the following topics do you include as part of counseling to patients presenting with unilateral SSNHL*? "Check all that apply"			
Possible causes (correct answer)		54	64.3
Available treatment options and associated risks/benefits (correct answer)		41	48.8
Impact on Quality of Life (correct answer)		40	47.6
Rehabilitation options (e.g., hearing aids) (correct answer)		38	45.2
I do not counsel patients presenting with SSNHL as I am rarely certain of the diagnosis upon initial presentation demographic questions		30	35.7
Knowledge Score (lowest possible score = 0, maximum possible score = 17)			
Minimum		3	
Maximum		17	
Mean		10.89	
Standard deviation		3.24	
*Sudden sensorineural hearing loss			

**Figure 3:**
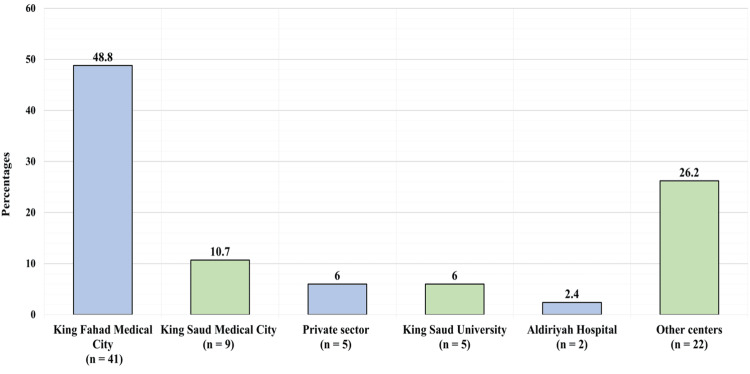
The hospital in which you primarily practice takes place

Figure 3 shows the knowledge level toward sudden sensorineural hearing loss. Twenty one (25%) of the participants had a low knowledge level (less than 50% of the total score) (a score of eight or less), 34 (40.5%) had a moderate knowledge level (between 50% and 75% of the total score) (a score between 9 and 12), and 29 (34.5%) had a high knowledge level (higher than 75% of the total score) (a score of 13 or higher). 

**Figure 4:**
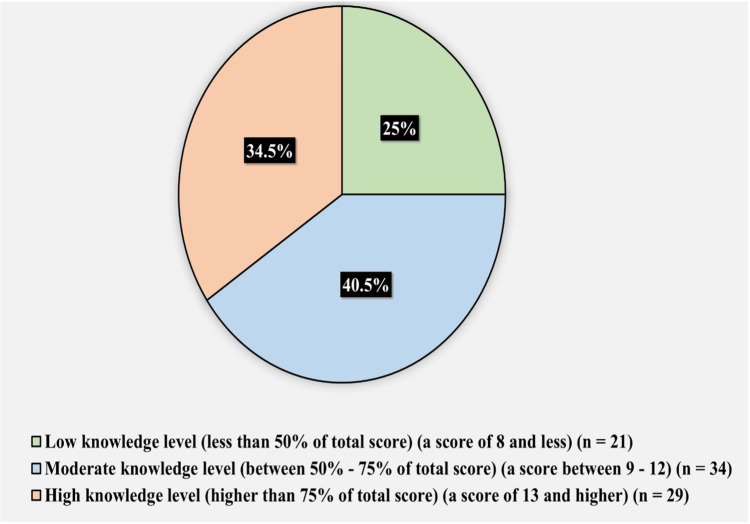
Knowledge level toward sudden sensorineural hearing loss

For the counseling of unilateral SSNHL patients, 54 physicians (64.3%) selected the topic of some potential reasons for hearing loss; 41 physicians (48.8%) preferred to discuss available therapy options and their associated risks and advantages; 40 physicians (47.6%) aimed to demonstrate the influence of hearing loss on the quality of life; 38 physicians (45.2%) included rehabilitation options like hearing aids; however, 30 physicians (35.7%) expressed that they did not want to initially counsel patients presenting with SSNHL due to the uncertainty of the disease (Figure 4).

## Discussion

The study evaluated the diagnostic and management approaches for SSNHL among family physicians in Riyadh, Saudi Arabia. Our research shows that even though family doctors knew SSNHL was a medical emergency that needed to be treated immediately, most of them could not recognize it immediately. When faced with indistinguishable SNHL, a crucial first step in SSNHL therapy that may affect the outcome of patients is quickly determining whether the loss is of the conductive or sensorineural kind, which will differ in the treatment of both cases. Fewer participants quickly made this determination. Only a small percentage of the participants could accurately define SSNHL in 72 hours; however, most of the participants couldn’t recognize the golden period. When faced with expected unilateral SSNHL, most primary care physicians who participated in our study made the right referral decisions, which is the right choice to make in such cases if nothing could be provided. While a tiny minority ordered unnecessary tests.

14.4% and 8.6% of participants were prescribed MRI and CT scans, respectively. However, both are not necessary for the initial evaluation of SSNHL (according to the Clinical Practice Guidelines (CPG) and the Canadian Society of Otolaryngology-Head and Neck Society, founded by the American Academy of Otolaryngology-Head and Neck Surgery Foundation, AAO-HNSF) for sudden hearing loss. A study in Saudi Arabia revealed that MRI scans often yield no abnormalities. Given their high rate and little yield of aberrant findings, MRIs may only be occasionally advised as the initial screening tool, and their regular usage should and might be curtailed to offset medical expenses [30]. The posterior cochlea is where pathology is detected by CT scans. It is not advisable to expose the patient to radiation. It is preferable to use gadolinium-enhanced MRI to image the brain, brainstem, and internal auditory meatus [30-31].

Referral doctors must be able to recognize SSNHL at the initial visit, particularly the ruling out of conductive hearing loss (CHL), to make an urgent ENT referral. Making the distinction between CHL and sensorineural hearing loss (SNHL) is a vital initial move for physicians dealing with unexpected hearing loss, according to the AAO-HNSF. This is because each requires a distinct therapy [30]. Using a TFT, particularly the Weber and Rinne tests, is the preferred method to discriminate between CHL and SNHL in the primary inspection of unilateral sudden hearing loss, and this was the case with 75% of the study participants [31-33]. The Weber test is extremely sensitive to the diagnosis of afflicted ears in SSNHL patients and can distinguish between SSNHL and CHL [34]. In the case of unilateral sensorineural hearing loss, the Rinne test results can support the Weber test findings by establishing no evidence of CHL within the afflicted ear [35].

Establishing that a sudden hearing loss is sensorineural also justifies primary care doctors starting to administer corticosteroids without first consulting an otolaryngologist, boosting the possibility that the patient may recover their hearing. Only 17.2% of participants reported administering corticosteroids for verified unilateral SSNHL. This figure suggests that not all participating primary care providers are aware of the potential benefits of corticosteroids in the initial management of SSNHL. However, the commencement of the corticosteroid therapy and the immediate referral to an ENT expert depend on the perception of the referral physician for SSNH, even though a prior study and analysis of the available literature revealed no statistically significant advantages associated with their use [36]. The majority of the physicians did not prescribe any treatment for SSNHL because they may believe that it can resolve spontaneously, as literature also reveals that 32-65% of cases recover spontaneously [12].

PHPs should consider TFTs, coupled with an otoscopy and extensive medical history, while making this choice. Our findings could be explained by the fact that the participating family doctors stated that they do not frequently encounter patients with sudden hearing loss to be able to do such tests regularly and sustain their skills. It, therefore, makes it more challenging to treat. In this situation, the doctor can substitute the Hum test. It is a Weber substitute that is simple yet efficient and has no need for a tuning fork. Despite the confirmed diagnosis, 45.3% (n=48) of the participating doctors believed that family physicians shouldn’t recommend any therapy for SSNHL. A similar survey study in Canada revealed the same results [2]. Our study cannot clearly explain their stance, but they may be worried about the systemic therapy's possible adverse side effects. There is currently a dearth of research examining how PCPs in Saudi Arabia treat patients with sudden hearing loss.

Limitations of the study

Given the limited sample size, the exclusion of other healthcare facilities, and the fact that most of the respondents have fewer than five years of relevant experience. It reveals that not all responses necessarily indicate the expertise and management techniques used by all family physicians in Riyadh.

## Conclusions

Given that SSNHL is a medical concern needing urgent care, many PCPs would make the proper diagnostic and therapeutic recommendations in dealing with SSNHL. Corticosteroids, the standard of care for initial treatment for SSNHL, would be prescribed by most participants, but not all complied. TFTs are a rapid and reliable way to differentiate between CHL and SNHL, but study participants rarely used them. Additionally, the majority of respondents expressed reliability when interpreting TFT results. Variability in first-line providers' handling of SSNHL may occur due to the absence of clear evidence-based standards for its therapy. In order to enhance conformity to standards based on evidence in the management of SSNHL, the study highlights the necessity for the establishment of precise recommendations.

## Appendices

Questionnaire questions assessing trends in SSNHL management

1. According to your definition, sudden sensorineural hearing loss (SSNHL) is defined as hearing loss that can develop over a period of: a. Less than 24 hours b. 48 hours c. 72 hours d. 7 days

2. Which of the following referrals do you make upon presentation of suspected unilateral SSNHL? Check all that apply. a. Lab work b. CT c. MRI d. Audiological evaluation e. Otolaryngology consultation f. Neurology consultation g. Emergency Department

3. In your practice, does unilateral, sudden-onset hearing loss warrant urgent referral for audiological testing? a. Yes b. No

4. In your practice, does unilateral SSNHL warrant urgent referral to otolaryngology? a. Yes b. No

5. When presented with unilateral, acute, or sudden-onset hearing loss, do you attempt to differentiate between conductive and sensorineural hearing loss? a. Yes b. No 6. Do you use tuning fork tests to differentiate between conductive and sensorineural hearing loss? a. Yes b. No 7. Do you feel confident in administering and interpreting the results of tuning fork tests to differentiate between conductive hearing loss and sensorineural hearing loss? a. Yes b. No 8. Do you feel comfortable interpreting a formal audiogram to differentiate between a conductive hearing loss and sensorineural hearing loss? a. Yes b. No 9. When presented with unilateral, sudden-onset hearing loss, which of the following do you use to inform management decisions? Check all that apply. a. Otoscope b. Case history c. Tuning fork test(s) d. Audiological evaluation e. Lab work 10. As a family physician, which of the following pharmacologic agents do you prescribe as treatment when presented with suspected unilateral SSNHL, prior to confirmation with audiological testing? Check all that apply. a. Corticosteroids b. Antivirals c. Thrombolytics d. Vasodilators e. Other (e.g., antibiotics) f. None of the above g. I do not prescribe any pharmacologic agents when presented with suspected SSNHL

11. As a family physician, which of the following pharmacologic agents do you prescribe as treatment when presented with confirmed, unilateral SSNHL? Check all that apply. a. Corticosteroids b. Antivirals c. Thrombolytics d. Vasodilators e. Other (e.g., antibiotics) f. None of the above g. Family physicians should not prescribe any treatment for SSNHL

12. Which of the following topics do you include as part of counseling to patients presenting with unilateral SSNHL? Check all that apply a. Possible causes b. Available treatment options and associated risks/benefits c. Impact on Quality of Life d. Rehabilitation options (e.g., hearing aids) e. None of the above f. I do not counsel patients presenting with SSNHL, as I am rarely certain of the diagnosis upon initial presentation

*Demographic Questions* 13. Years in practice a. Less than 5 years b. 5 - 10 years c. 11 - 15 years d. 16 - 20 years e. More than 20 years

14. The setting in which you primarily practice would be considered: a. Solo practice b. Walk-in clinic c. Urgent Care or Emergency Department d. Non-Academic Group or Team e. Academic Group or Team 15. In the past 6 months, the number of patients presenting to you with complaints of unilateral, acute, or sudden-onset hearing loss, not as a result of cerumen impaction: a. Fewer than 5 b. 5-10 c. 11-15 d. 16-20 e. More than 20

16. In the past 12 months, the typical wait time for your patients to be seen by an otolaryngologist, referred for unilateral, sudden-onset hearing loss: a. 1 week or less b. 1-4 weeks c. 1-3 months d. 3-6 months e. Greater than 6 months f. Greater than 12 months g. I don’t know

17. The hospital in which you primarily practice takes place: A. King Fahad Medical City B. King Saud Medical City C. Aldiriyah Hospital D. Private sector E. King Saud University F. Imam Mohammed ibn Saud Islamic University
 
